# An open-source transparent microelectrode array

**DOI:** 10.1088/1741-2552/ac620d

**Published:** 2022-04-13

**Authors:** Isaac A Weaver, Austin W Li, Brenda C Shields, Michael R Tadross

**Affiliations:** Department of Biomedical Engineering, Duke University, Durham, NC, United States of America

**Keywords:** transparent multi-electrode array, electrophysiology, glass indium tin oxide (ITO) electrodes, photometry, optogenetics, printed circuit board pre-amplifier, open source

## Abstract

**Objective.:**

The micro-electrode array (MEA) is a cell-culture surface with integrated electrodes used for assays of electrically excitable cells and tissues. MEAs have been a workhorse in the study of neurons and myocytes, owing to the scalability and millisecond temporal resolution of the technology. However, traditional MEAs are opaque, precluding inverted microscope access to modern genetically encoded optical sensors and effectors.

**Approach.:**

To address this gap, transparent MEAs have been developed. However, for many labs, transparent MEAs remain out of reach due to the cost of commercially available products, and the complexity of custom fabrication. Here, we describe an open-source transparent MEA based on the OpenEphys platform.

**Main results.:**

We demonstrate the performance of this transparent MEA in a multiplexed electrical and optogenetic assay of primary rat hippocampal neurons.

**Significance.:**

This open-source transparent MEA and recording platform is designed to be accessible, requiring minimal microelectrode fabrication or circuit design experience. We include low-noise connectors for seamless integration with the Intan Technologies headstage, and a mechanically stable adaptor conforming to the 24-well plate footprint for compatibility with most inverted microscopes.

## 1. Introduction

Electrophysiology with a micro-electrode array (MEA) is a foundational technique for the study of electrically excitable cells and tissues. MEAs offer a non-invasive means to detect the extracellular field potential generated by an action potential from a single neuron or myocyte. Extracellular electrophysiology offers millisecond temporal fidelity, stability of recording for days to weeks, and scalability to devices containing hundreds to thousands of electrodes [1–3]. Often, however, electrophysiological measurements alone do not tell the full story. Optical reporters including genetically encoded indicators for calcium and biochemical events, and a myriad of optical stimulation methods to induce electrical and light-gated biochemical signals are increasingly needed [4–12]. Ideally, these optical methods would be used in conjunction with electrophysiological recordings to address specific hypotheses in cellular physiology and pharmacological screens. However, many available electrodes are opaque and must be mounted on a stand-alone recording platform, restricting experimental flexibility.

Although transparent cell-culture recording systems that integrate electrophysiological recordings with optical access have been developed, most are expensive or technically challenging to manufacture and implement. OpenEphys has changed the electrical-recording landscape by significantly reducing the financial barrier to entry [13]. However, existing devices are primarily geared towards *in vivo* recording, with little focus on *in vitro* recording. To address this gap, we developed an open-source transparent MEA that can interface with the OpenEphys platform and mount on a standard inverted microscope. In this first implementation, we describe a system consisting of a transparent 64-channel MEA, preamplifier printed circuit board (PCB), and microscope mount enabling compatibility with any standard 24-well-plate stage insert. We optimized the design to enable compatibility with academic fabrication facilities, to increase accessibility, minimize cost, and streamline fabrication. We provide detailed protocols for fabrication of the transparent microelectrode array (photolithography mask, based on [14], and process follower instructions), the preamplifier circuit board (PCB design files), the acrylic PCB mount (Computer Aided Design—CAD file), and the customizable microscope baseplate insert (CAD files).

## 2. Materials and methods

### 2.1. Overview of microelectrode array development

Our goal was to develop a transparent MEA that combines high performance with low cost. We sought to keep raw-material cost below $10 per device and maintain compatibility with academic cleanroom facilities. To keep with these goals, MEA fabrication was designed to use basic microelectronic photo-lithographic techniques [15]. For transparency, glass was used as the base substrate. For patterning the electrical components of the array, we considered many materials that would maintain transparency. We excluded certain materials, such as graphene [16], which require advanced fabrication, and thus may pose a challenge for open-source assembly. Conversely, we excluded insulator materials such as nitride or oxide owing to the specialized equipment required to deposit such materials and the additional steps needed to pattern them [17]. We ultimately selected indium tin oxide (ITO) as the conductor and SU-8 as the insulator due to their transparency, electrical conductivity, biocompatibility, and ease of fabrication [18, 19].

### 2.2. Transparent microelectrode array design, fabrication, and packaging

**Figure 1.**
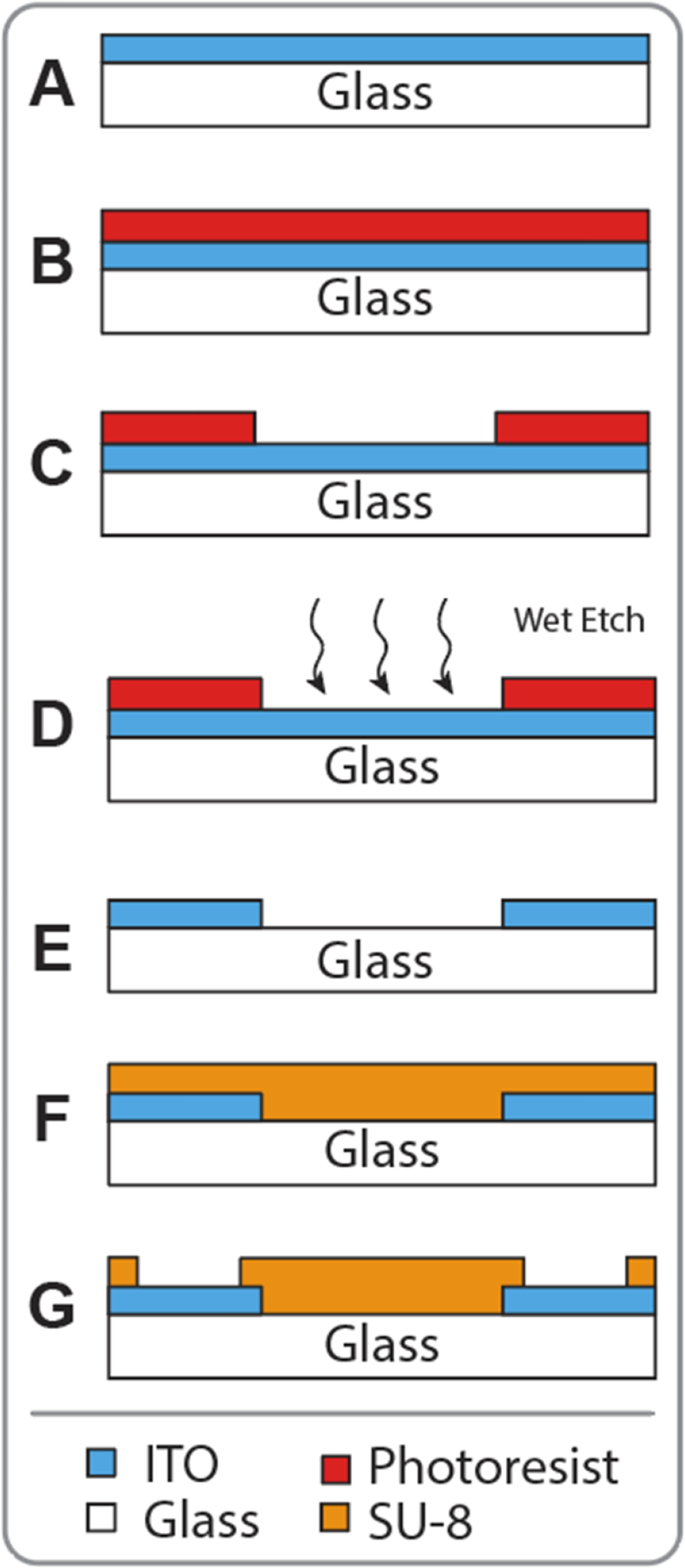
Illustration of fabrication steps: (A) cross section of base substrate (ITO coated glass wafer); (B) photoresist deposition; (C) photoresist patterning; (D) ITO patterning via wet etch; (E) photoresist stripped following wet etch; (F) SU-8 deposition; (G) SU-8 patterning and hard bake.

Fabrication was performed at the Shared Instrumentation Materials Facility cleanroom at Duke University. ITO-coated borosilicate glass wafers (100 mm diameter, ∼10 Ω sq^−1^, University Wafer) were used as base substrates (figure 1(A)). P20 adhesion promoter (Shin-Etsu Micro Si) was flash deposited while each wafer was spinning on the spin coater (Headway) then Shipley S1813 positive photoresist (Kayaku Advanced Materials) was deposited and spun at 3000 rpm for 30 s, and baked for 1 min at 115 °C (figure 1(B)). Wafers were exposed on a mask aligner (MA/BA6, Karl Suss) for a 120 mJ cm^−2^ ultraviolet (UV) exposure developed in MIF 319 developer (Kayaku Advanced Materials) for 60 s with mild agitation, and then thoroughly cleaned with dionzed (DI) water (figure 1(C)). After inspecting the lithography of each wafer, the exposed ITO was etched by submerging samples into a strongly acidic solution (4:2:1 HCl:H_2_O:HNO_2_ by volume); etch time varied from 3 to 5 min depending on the potency of the acidic solution, which decreased exponentially over time due to the reaction of HCl and HNO_2_ to form *aqua regia* (figure 1(D)). To ensure that the ITO had been etched sufficiently, the sheet resistances of etched areas were measured using a four-point probe and confirmed to be at least 100 × 10^6^ Ω sq^−1^ before proceeding. After etching, remaining photoresist was removed by sonicating each sample in acetone for 10 min, exposing the patterned ITO conductive material (figure 1(E), blue).

SU-8 (Kayaku Advanced Materials) insulator was deposited onto the samples (figure 1(F)) by spin coating at 2000 rpm for 30 s (for a thickness of 2 *μ*m),then baked at 65 °C for 1 min, followed by 95 °C for 1 min. This temperature ramp was important as it minimized the mechanical stress applied to the SU-8 layer, which can crack if heated too quickly. The wafers were then allowed to cool slowly to ambient room temperature. To prepare the wafers for exposure, opaque wafer dicing tape (Ultron Corporation) was placed on the back of the wafers to prevent unwanted reflection of UV light during exposure of 100 mJ cm^−2^. The exposed samples were then baked in the same stepwise fashion as previously described before developing for 90 s with fast agitation. The samples were cleaned with isopropanol, inspected using green light, and baked for 30 min at 150 °C. Thus, the insulator was removed from the electrode pads (i.e. the recording surface) while retaining insulator over the wiring (figure 1(G)).

After baking, the wafers were inspected to ensure that all 20 *μ*m diameter pads had been fully cleared of SU-8 insulator. The wafers were then diced into four MEA devices. For the culture well, oval chambers (6 mm tall × 23 mm long × 16 mm wide with 2 mm wall thickness) were laser cut from an acrylic sheet (8560K358, McMaster Carr). After cutting, the wells were cleaned thoroughly to remove any charring. For final assembly of each device, the bottom of an acrylic well was coated in a thin layer of polydimethylsiloxane (PDMS) polymer (761036, Millipore Sigma) and center-placed atop a MEA device. The assembled device is baked at 80 °C for 1 h to cure the PDMS. The final device has a 24 mm × 40 mm footprint (figure 3(B)).

### 2.3. PCB design and assembly

**Figure 2.**
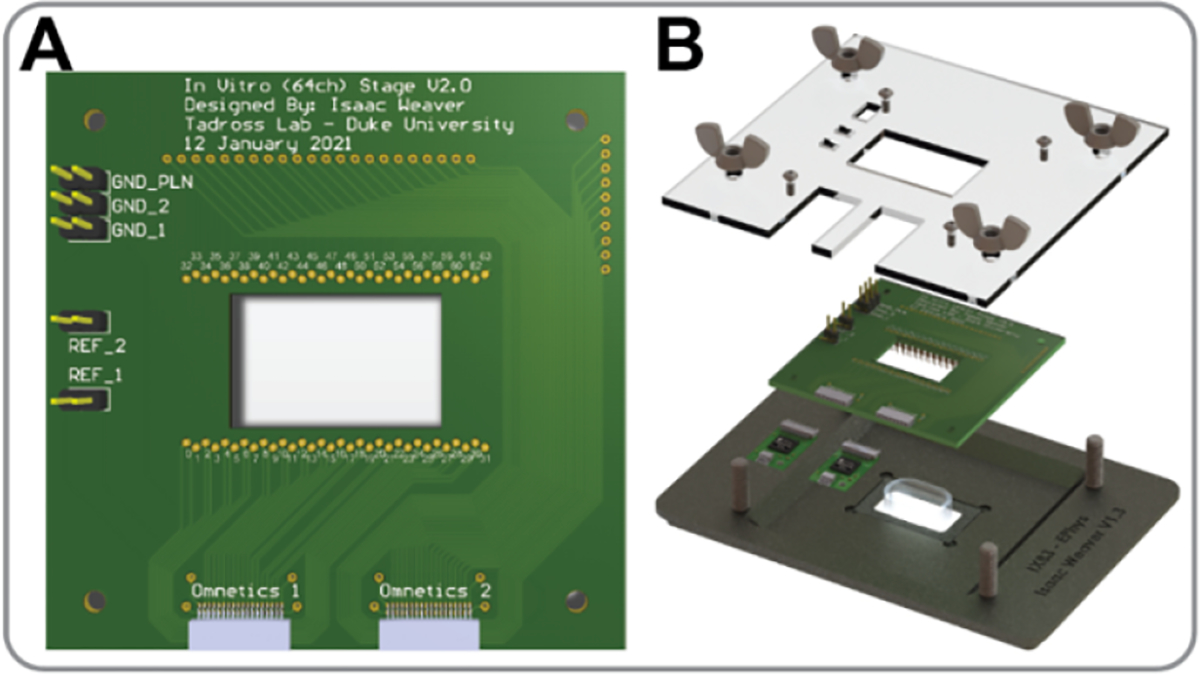
Render of MEA interface: (A) PCB with assembled components; (B) PCB with clamshell incorporation into the inverted microscope stage attachment with MEA.

The PCB was designed as an interface between the MEA and the OpenEphys acquisition system. Our goals were to minimize electrical noise, maximize mechanical stability with repeated use, and to make it easy to quickly connect or remove a sample. To achieve the mechanical goals, the PCB uses spring-loaded pins to make contact with the MEA, enabling reliable contact and easy loading of MEAs with minimal physical wear on the PCB (figure 2(A)). For compatibility with the OpenEphys system, the 64 channel board connects directly to two 32-channel RHD 32 headstages (C3314, Intan Technologies) via industry-standard Omnetics connectors, immobilized with cyanoacrylate glue for physical stability. For electrical considerations, we incorporated a large ground plane that can be connected to a surrounding Faraday cage.

### 2.4. Inverted microscope stage attachment

Ease of integration was important when designing the interface with existing inverted microscopes. Here we detail two versions of the stage interface: the first designed specifically to fit into an ASI AZ-2000FT stage, and the second to fit into a 24-well plate cut out for compatibility with most microscopes. Both designs use a clamshell base to interface with the microscope stage, and a clamshell top affixed to the PCB. The design allows an MEA to be easily inserted within the clamshell, ensuring reliable contact between the MEA and spring-loaded pins of the PCB. A cut-out in the base allows for optical access from below.

To assemble the system, the clamshell base is attached to the microscope stage, then the PCB is bolted to the clamshell top. Next, an MEA is centered in the clamshell base cut-out, and the clamshell top tightened down using wing nuts. The RHD 32 headstages are then attached to the board and a silver chloride reference electrode is added to the well and connected to the reference pin on the board (figure 2(B)).

### 2.5. MEA electropolymerization

**Figure 3.**
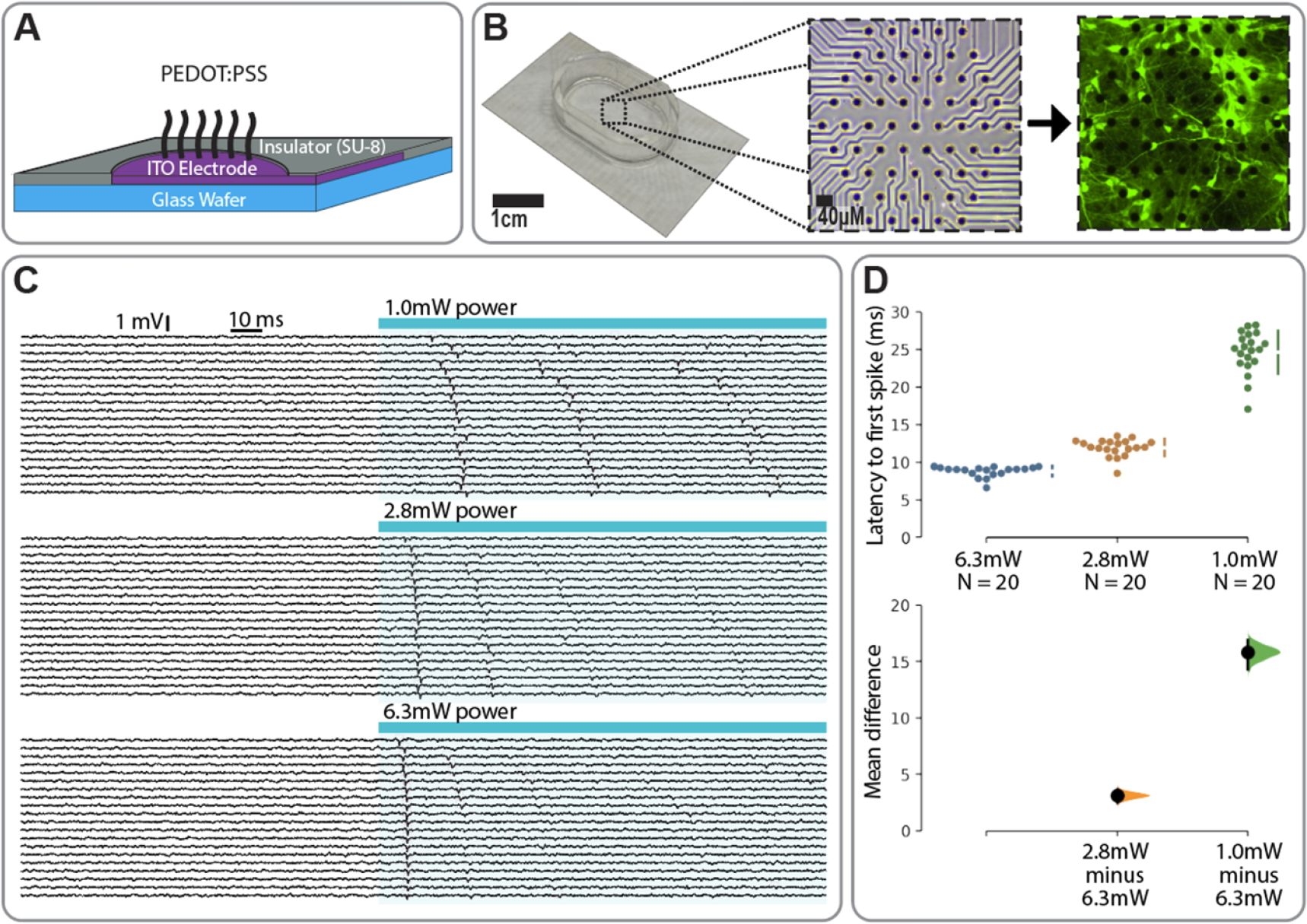
Transparent MEA device and recording: (A) cross section of device showing material layers and conductive polymer coating (PEDOT:PSS); (B) photo of MEA (left), zoomed insert showing PEDOT:PSS coated electrode pads after deposition (middle). Fluorescence image (right) showing the same device weeks later with cultured neurons expressing GCaMP6s. Green intensity represents the delta-F in response to optogenetic illumination (maximum minus minimum of GCaMP6s signal). (C) Electrophysiological recordings showing millisecond-precision extracellular spike waveforms in response to optogenetic stimulation at various optical power intensities; (D) analysis of latency to first spike as a function of illumination intensity.

To increase conductivity of the 20 *μ*m diameter MEA pads, we polymerized PEDOT:PSS (poly ethylenedioxythiophene:poly styrene sulfonate) via standard protocols [20]. Briefly, a solution of 10*μ*M EDOT and 100 *μ*M PSS was prepared in deionized H_2_O, applied to the MEA surface, and connected to a Keithley 2450 source measure unit (Keithley 2450-EC, Tektronix, Inc.) such that the anode corresponded to the ITO pads of the MEA, and the cathode was a silver chloride wire in solution [21]. Electropolymerization was driven by holding a potential of 1 V for 10 min (figures 3(A) and (B)). The bare ITO 20 *μ*m diameter MEA pads were found to have a measured impedance of 1.15 ± 0.19 MΩ at 1 kHz. After polymerization, the PEDOT coated ITO electrode sites were found to have a measured impedance of 92.75 ± 2.97 KΩ at 1 kHz. These values are consistent with previous studies[22].

### 2.6. Device preparation and primary neuronal culture

Devices were coated with high molecular weight poly-d-lysine (PDL) to improve neuronal attachment and biocompatibility [23]. One milliliter of PDL (P7405, Sigma) diluted to 0.5 mg ml^−1^ was added to the well and incubated for 1 h at room temperature with mild agitation. The well was washed ten times with water, followed by an ethanol wash, then left to dry.

Primary hippocampal neurons were isolated from postnatal rat pups using established protocols [24], then electroporated (V4SP-3096, LONZA) with genetic constructs containing ChR2-mScarlet (opsin that drives action potentials in response to light fused to a red fluorescent protein) and GCaMP6s (green fluorescent calcium indicator) [25]. Approximately one million cells were seeded onto each device and flooded with NbActiv4 media (NB4, BrainBits). Samples were maintained in a 37 °C, 5% CO_2_ incubator for 2 weeks, with half of the media replaced at DIV3 then weekly thereafter. Mature cells were healthy on the surface of the devices (figure 3(B), right).

### 2.7. Data acquisition and analysis

Samples were prepared for recording by gradually replacing NB4 culture media with Tyrode’s solution to minimize shear stress. The MEA devices were then mounted onto an inverted fluorescent microscope (IX83 with 10× APO air objective, Olympus Lifescience) using the ASI-stage clamshell design and connected via dual Intan RHD 32-channel headstages to the OpenEphys acquisition system. Samples were centered on the stage and focused under at 580 nm excitation (mScarlet). For optogenetic induction of action potentials, samples were illuminated with 470 nm blue light. Electrical recordings were collected in the OpenEphys software environment and analyzed offline in Matlab.

## 3. Results

Our system allowed for straightforward imaging of fluorescent proteins, including GCaMP6s, with optical fidelity on par with that seen on glass coverslips. For example, figure 3(B) shows the GCaMP6s response to optogenetic stimulation, with dozens of responsive neurons in a field of view. While the number of detectible neurons and subcellular resolution afforded by GCaMP6s is remarkable, a known limitation is the slow ∼seconds-long temporal resolution. Thus, a key feature of the transparent MEA is the ability to pair optical tools with millisecond-precision electrical recording of neuronal spikes.

Figure 3(C) shows an example in which we examined the impact of optogenetic illumination with long (2 s) blue light over a range of intensities (1–6.3 mW). In these experiments, we blocked synaptic transmission with a combination of drugs (10 *μ*M gabazine to block inhibitory synapses; 10 *μ*M NBQX—2,3-dioxo-6-nitro-7-sulfamoyl-benzo[f]quinoxaline—and 10 *μ*M CPP—3-(2-Carboxypiperazin-4-yl)propyl-1-phosphonic acid—to block excitatory synapses), thus isolating the optogenetic component of activity (figure 3(C)). As a simple demonstration of the platform, we measured the latency to first spike at different illumination intensities (figure 3(D)). As expected, we found that brighter illumination reduced latency to first spike, with a latency of 24.6 ms at 1 mW power, 11.9 ms at 2.8 mW power, and 8.8 ms at 6.3 mW power (unpaired mean difference between 6.3 mW power and 2.8 mW power of 3.12 ms and an unpaired mean difference between 6.3 mW power and 1.0 mW power of 15.8 ms [26]).

Beyond latency, we also observed a qualitative tendency for low-intensity illumination to produce multiple spikes over time, where as high-intensity illumination tended to produce a single spike. This is consistent with optogenetic-induced depolarization block induced by high-intensity illumination. Interestingly, we observed that GCaMP signals continued to rise over time despite the induction of depolarization block, suggesting a direct recruitment of voltage-gated calcium channels by the graded optogenetic depolarization. These observations highlight the potential for discordance between calcium signals and neuronal spikes [25], and emphasize the importance of obtaining ground-truth electrophysiological recordings of spikes, particularly under conditions of graded depolarization.

## 4. Discussion

The combination of electrical and optical access in the transparent MEA offers versatility with many applications—including optogenetic stimulation, optical sensors for calcium, and other second messengers such as cAM—3’,5’-cyclic adenosine monophosphat. While commercially available transparent MEAs exist, they are expensive and proprietary, raising the barrier to entry for many academic labs, and limiting flexibility of the hardware and software. Here, we present an open-source alternative to address issues of accessibility, cost, and flexibility.

With regard to data acquisition and analysis, the use of Intan headstages and the OpenEphys platform ensures that all software components are open-source, fully customizable, and supported by a growing community of academic developers. With regard to accessibility, we tailored the open-source MEA design to satisfy the majority of biological applications, thus minimizing the need for engineering expertise, and maximizing accessibility to biologists. We provide all necessary design and process files under an open-source license, and all fabrication steps are within the capacity of academic fabrication facilities.

For users with unique hardware needs, we made design choices to facilitate user-specific customizations that can integrate with the base design. For example, the PCB design was via CircuitMaker (Altium Limited) software, a free program that provides unlimited access to end-user modifications. We have similarly included the files for both stage adapter designs, enabling customization for unique microscopes. The MEA mask is also customizable, allowing adjustment of pad diameter and placement. For advanced microelectromechanical systems applications, the device is compatible with fabrication via photolithography, enabling adaptation of the transparent MEA in combination with a wide range of integrated sensor platforms. For example, the borosilicate glass substrate and SU-8 insulator should facilitate bonding to microfluidic chip materials including PDMS. Such integration of microfluidics with optics and electrical access could enable advanced platforms for drug discovery and therapeutics.

## Data Availability

The data that support the findings of this study are openly available at the following URL/DOI: https://gitlab.oit.duke.edu/mrt31/transparent_mea/.
